# Elevated Serum Levels of Soluble ST2 Are Associated With Plaque Vulnerability in Patients With Non-ST-Elevation Acute Coronary Syndrome

**DOI:** 10.3389/fcvm.2021.688522

**Published:** 2021-07-22

**Authors:** Guqing Luo, Yuxuan Qian, Xincheng Sheng, Jiateng Sun, Zhinan Wu, Fei Liao, Qi Feng, Yan Yin, Song Ding, Jun Pu

**Affiliations:** ^1^Department of Cardiology, School of Medicine, Renji Hospital, Shanghai Jiaotong University, Shanghai, China; ^2^Department of Radiology, School of Medicine, Renji Hospital, Shanghai Jiaotong University, Shanghai, China

**Keywords:** coronary computed tomography angiography, coronary plaque, plaque vulnerability, soluble ST2, non-ST elevation acute coronary syndromes

## Abstract

**Background:** Recent studies have suggested that soluble suppression of tumorigenicity-2 (sST2), an inflammation-related protein receptor, is associated with atherosclerotic diseases. This study aimed to investigate the potential predictive value of sST2 on plaque vulnerability by assessing whether elevated serum levels of sST2 are associated with vulnerable plaque features in patients with non-ST-elevation acute coronary syndrome (ACS).

**Methods:** A total of 120 patients with non-ST-elevation ACS (167 lesions) were prospectively enrolled and evaluated by standard coronary computed tomography angiography (CCTA) and coronary angiography in this study. Serum sST2 levels were measured by ELISA (Presage^®^ ST2 Assay Kit, Critical Diagnostics), and semiautomated software (QAngioCT, Medis) was used to quantify coronary plaques.

**Results:** The included patients were divided into 4 groups by serum sST2 level quartiles. Volumetric analysis of the whole lesion revealed that patients with higher sST2 levels had a larger absolute necrotic core (NC) volume (Quartile 4 vs. Quartile 1, 86.16 ± 59.71 vs. 45.10 ± 45.80 mm^3^, *P* = 0.001; Quartile 4 vs. Quartile 2, 86.16 ± 59.71 vs. 50.22 ± 42.56 mm^3^, *P* = 0.002) and a higher NC percentage (Quartile 4 vs. Quartile 1, 35.16 ± 9.82 vs. 23.21 ± 16.18%, *P* < 0.001; Quartile 4 vs. Quartile 2, 35.16 ± 9.82% vs. 22.50 ± 14.03%, *P* < 0.001; Quartile 4 vs. Quartile 3, 35.16 ± 9.82% vs. 25.04 ± 14.48%, *P* < 0.001). Correlation analysis revealed that serum sST2 levels were positively correlated with the NC (*r* = 0.323, *P* < 0.001) but negatively correlated with dense calcium (*r* = −0.208, *P* = 0.007). Furthermore, among those with plaque calcification, patients with spotty calcification exhibited higher serum sST2 levels than those with large calcification (26.06 ± 16.54 vs. 17.55 ± 7.65 ng/mL, *P* = 0.002). No significant differences in plaque components at the level of the minimal lumen area (MLA) were found among the groups.

**Conclusions:** Serum sST2 levels were correlated with different coronary plaque components in patients with non-ST-elevation ACS. A higher serum level of sST2 was correlated with plaque vulnerability.

**Clinical Trial Registration:** www.ClinicalTrials.gov, identifier: NCT04797819.

## Introduction

Rupture of vulnerable plaques and subsequent thrombosis are the main triggers of acute coronary syndrome (ACS), and researchers are becoming increasingly interested in the early identification of vulnerable plaques. Standard intracoronary imaging methods, such as intravascular ultrasound (IVUS) and optical coherence tomography (OCT), are used to quantify the distribution and severity of coronary plaques but are limited by their invasive features. Coronary computed tomographic angiography (CTA) is a sensitive and non-invasive modality widely used for the diagnosis of coronary artery disease. QAngioCT can quantitatively analyze stenosis, plaque burden, and specific intraplaque components of coronary CTA (CCTA) images by obtaining 3-dimensional centerline of coronary artery and reconstructing coronary artery volume via a fast vessel-tracking algorithm (1, 2). Furthermore, several studies supported the feasibility of non-invasive quantitative CCTA (QCCTA) via QAngioCT software to assess plaque burden and plaque components when compared to quantitative coronary angiography (QCA) or virtual histology (VH)-IVUS analysis (1–3).

Inflammation plays an essential role in the pathogenesis of plaque vulnerability (4). sST2, a soluble form of ST2, can modulate the inflammatory response and exert proinflammatory effects when secreted into the circulation (4, 5). Elevated serum levels of sST2 have been observed in patients with several inflammatory and autoimmune diseases, including inflammatory bowel disease, asthma, and rheumatoid arthritis. Several studies have focused on the association between serum sST2 and cardiovascular diseases. Previous studies demonstrated that elevation of serum sST2 levels was associated with poor prognosis in patients with myocardial infarction (MI) or heart failure (HF) (6–8). An animal study has reported that administration of sST2 exacerbated atherosclerosis development in a mouse model (9). Consistent with that, a recent study by Zhang et al. showed that serum sST2 levels were elevated in patients with ACS, especially in those with complex lesions (4). In this study, we aimed to investigate the relationships between serum sST2 levels and CCTA-based plaque components in patients with non-ST-elevation ACS. We hypothesized that elevated serum sST2 level might be closely related to vulnerable plaque features, serving as a reliable sensor of coronary immune-inflammatory disorder and a simple indicator for coronary plaque vulnerability.

## Methods

### Study Population

**Figure 1:**
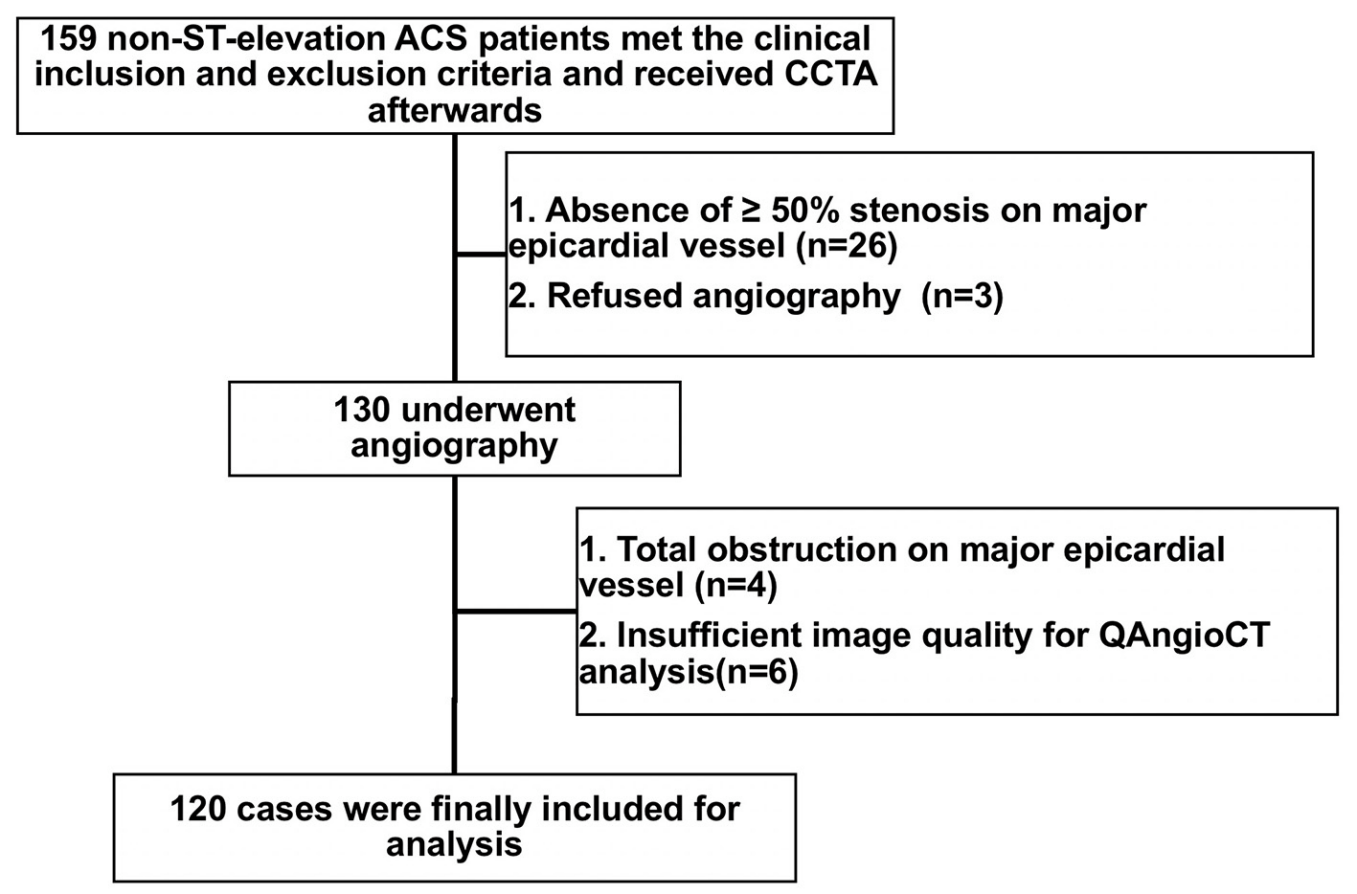
Flowchart of patient enrollment. ACS, acute coronary syndrome; CCTA, coronary computed tomography angiography.

Patients with non-ST-elevation ACS who required an immediate (<2 h) or early invasive strategy (<24 h) according to guidelines, including those who presented with hemodynamic instability or cardiogenic shock, life-threatening arrhythmias or cardiac arrest, mechanical complications, acute heart failure, dynamic ST or T wave changes, or a Global Registry of Acute Coronary Events (GRACE) score > 140, were excluded (10). In addition, subjects with a previous history of coronary artery bypass graft surgery or percutaneous coronary intervention (PCI), immune system disorder, cancer, acute/chronic infection, statin use within 3 months, atrial fibrillation, end-stage renal failure, or iodine-containing contrast allergy were excluded. Between January 2019 and December 2019, a total of 159 patients with non-ST-elevation ACS (non-ST-elevation myocardial infarction or unstable angina) aged 18–75 years who underwent CCTA were prospectively enrolled in this study. After CCTA, we also excluded patients with no significant (≥50%) stenosis of major epicardial vessels (*n* = 26) and those who refused subsequent angiography (*n* = 3). Among the 130 remaining patients who received angiography, those with total obstruction of major epicardial vessels (*n* = 4) or insufficient image quality for QAngioCT analysis (*n* = 6) were excluded. Finally, a total of 120 patients with 167 lesions were included in our study for the final analysis (Figure 1). The baseline features and cardiovascular risk factors of the study subjects were documented. This study was approved by the Institutional Review Board of Renji Hospital, and all subjects provided written informed consent.

### Serum sST2 and Other Biochemical Indicators Measurement

At admission, four milliliters of venous blood were collected from the antecubital veins of patients hospitalized for the angiography procedure into EDTA-containing tubes. The blood was centrifuged at 3,000 g for 5 min within 1 h of collection, and the serum was immediately separated and stored at −80°C for further testing. Serum sST2 levels were measured with a commercial ELISA kit (Presage^®^ ST2 Assay Kit, REF#BC-1065, Critical Diagnostics, San Diego, CA) according to the manufacturer's instructions. Following standard laboratory techniques, other serum biochemical indicators including alanine transaminase (ALT), serum creatinine (Scr), high-sensitivity C-reactive protein (hs-CRP), and low-density lipoprotein (LDL) were analyzed at hospital admission as well.

### Enhanced Coronary CTA

Enhanced coronary CT scans were obtained from all of the included patients on a 320-slice CT scanner (Aquilion ONE, Toshiba Medical Systems, Otawara, Japan). To optimize the imaging quality, oral metoprolol was administered to patients with a heart rate >75 beats/min prior to the CT scan. The tube voltage and current for each patient was determined by Toshiba integrated dose reduction technique (SureExposure 3D). Electrocardiograms were used for retrospective gating to eliminate motion interference. Imaging data were reconstructed at a slice thickness of 0.5 mm and a reconstruction interval of 0.25 mm. All the results were interpreted by radiologists with board certifications for cardiac CT interpretation.

### Plaque Characteristics Analysis

**Figure 2:**
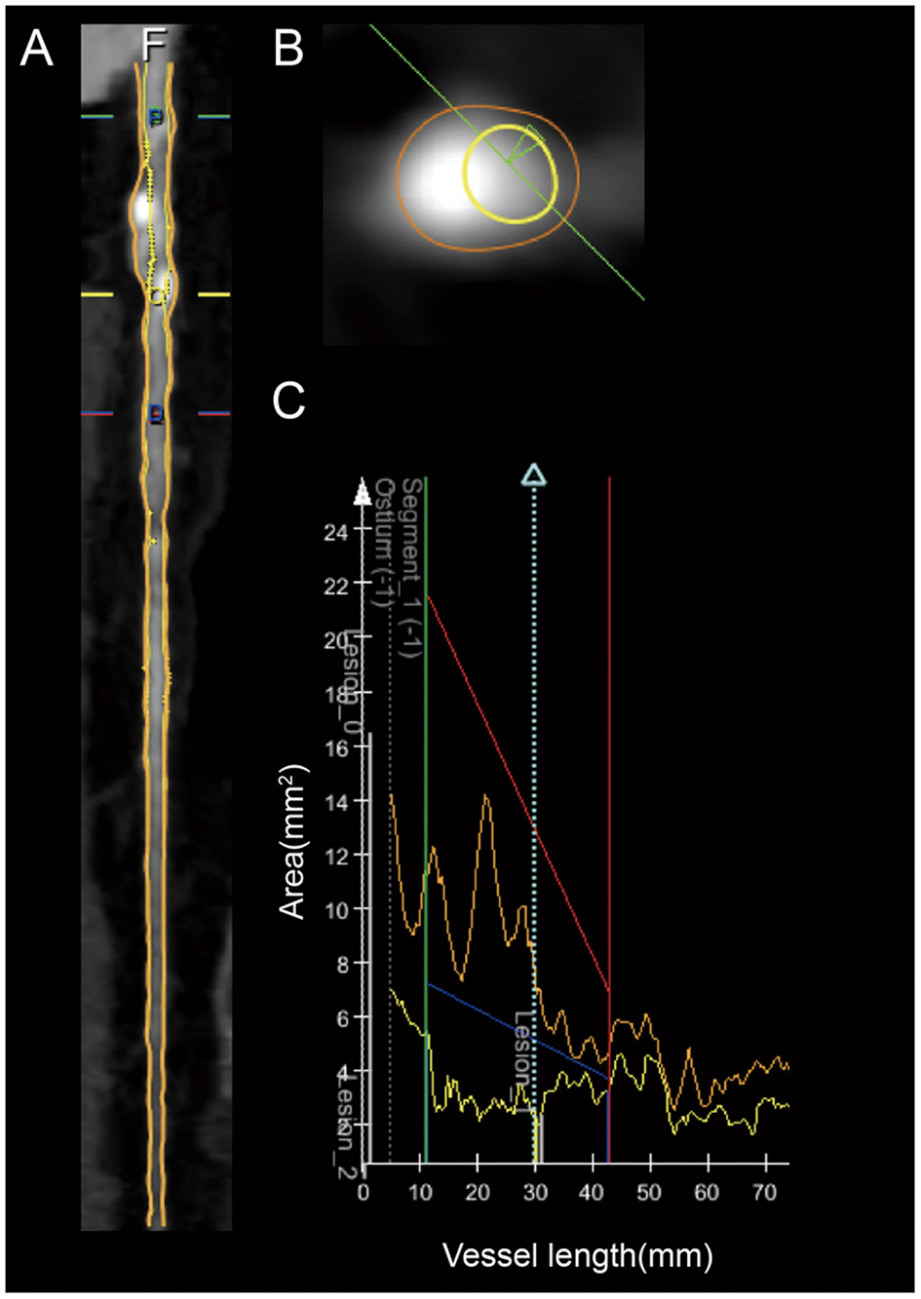
Example of the coronary plaque quantitative analysis of a lesion in the proximal LAD artery segment. **(A)** Longitudinal straightened multiplanar reconstruction, where “O” indicates the minimal lumen area. **(B)** Cross-sectional view of minimal lumen area. **(C)** Graph of the lumen and vessel area as a function of vessel length.

CCTA data were transferred to offline workstations. Plaque characteristics were semiautomatically analyzed using QAngioCT software (Medis^®^ QAngio CT V3.1, Medis Medical Imaging Systems, Leiden, the Netherlands). All lesions with a stenosis of ≥50% underwent quantitative analysis (11–13). Plaque characteristics were analyzed by two trained observers who were blinded to the clinical characteristics of the corresponding patients. QAngioCT software was used for the automated 3-dimensional reconstruction of the coronary artery volume to determine the contours of the vessel wall and lumen (Figure 2). Volumetric characterization of the plaque characteristics focused on the entire plaque volume under 3-dimensional reconstruction, while the cross-sectional characterization focused at the level of the minimal lumen area (MLA) (2, 14). And in our study, the level of the MLA was automatically identified using the lumen contours detected on coronary CTA (2). Different plaque components on CCTA were distinguished by Hounsfield unit (HU) values, and different cut-off values were available in previous studies, which were obtained by comparing coronary CTA with VH-IVUS or histological examination (15, 16). For the current study, a density of −30 ~ 75 HUs indicated necrotic core (NC), while a density of 76 ~ 130 HUs indicated fibrous fatty (FF), a density of 131 ~ 350 HUs indicated fibrous tissue (FT), and a density >351 HUs indicated dense calcium (DC) (14, 17). The eccentricity index and remodeling index were automatically calculated by QAngioCT software. Eccentricity index was calculated as (maximal plaque thickness minus minimal plaque thickness) divided by maximal plaque thickness (2, 18, 19). At the level of the minimal lumen area, the remodeling index was calculated by dividing the cross-sectional vessel wall area by the corresponding reference area. The cross-sectional reference area was determined in the normal-appearing reference area as close as possible to the respective coronary lesion (2, 18, 20). Spotty calcification was defined when <3 mm in size on curved multiplanar reformation images and 1-sided on cross-sectional images. Large calcification was defined as the calcification larger than spotty calcification (21, 22).

### Statistical Analysis

Statistical analyses were performed using IBM SPSS Statistics 23 (SPSS, Inc., Chicago, IL). Summary statistics of continuous data with symmetric distribution are expressed as the mean ± standard deviation (SD), while categorical data are expressed as counts (percentages). Comparison of continuous variables between two groups was performed using the independent sample *t*-test, while categorical variables were compared using the chi-squared test. Pearson's chi-square test was used to compare the constituent ratios and to assess the correlations between two continuous variables. *P* < 0.05 indicate statistical differences. Furthermore, threshold level of significance for differences among groups were adjusted for multiple comparisons by Bonferroni's correction. As 6 tests were performed among 4 groups, the differences were statistically significant when the observed *P*-values were less than the specified significance level (α) divided by the number of tests (K) = 0.05/6 <0.0084.

## Results

### Baseline Characteristics

Out of 120 patients, 167 lesions were analyzed in our study. Eighty-two patients had one lesion undergoing analysis, while 29 patients had two lesions and 9 patients had three lesions undergoing analysis. Patients were classified into 4 groups according to the serum sST2 level quartiles (sST2 < 14.5 ng/mL, Quartile 1; 14.5 ng/mL ≤ sST2 < 20.5 ng/mL, Quartile 2; 20.5 ng/mL ≤ sST2 < 25.9 ng/mL, Quartile 3; sST2 ≥ 25.9 ng/mL, Quartile 4). A comparison of the baseline characteristics is shown in Table 1. Patients with higher serum sST2 levels were older (Quartile 4 vs. Quartile 2, 70.27 ± 8.38 vs. 64.17 ± 8.91 ng/mL, *P* = 0.008). There were no statistically significant differences in the sex, BMI, heart rate, blood pressure, incidence of hypertension, hyperlipemia, diabetes mellitus, chronic obstructive pulmonary disease, known valvular disease, ALT level, Scr level, hs-CRP level, or eGFR among the groups. The lesion distributions across the main coronary arteries were not significantly different.

**Table 1:** Baseline characteristics.

	**Quartile 1 (*N =* 30) <14.5 ng/mL**	**Quartile 2 (*N =* 30) 14.5–20.5 ng/mL**	**Quartile 3 (*N =* 30) 20.5–25.9 ng/mL**	**Quartile 4 (*N =* 30) >25.9 ng/mL**	***P*****-value**					
					**Quartile 2 vs. Quartile 1**	**Quartile 3 vs. Quartile 1**	**Quartile 4 vs. Quartile 1**	**Quartile 3 vs. Quartile 2**	**Quartile 4 vs. Quartile 2**	**Quartile 4 vs. Quartile 3**
Age, yrs	65.10 ± 8.48	64.17 ± 8.91	65.60 ± 6.78	70.27 ± 8.38	0.679	0.802	0.021	0.486	0.008	0.021
Sex, M/F	20/10	24/6	21/9	22/8	0.250	0.786	0.581	0.380	0.549	0.779
BMI, kg/m^2^	25.15 ± 2.47	25.75 ± 4.05	24.66 ± 2.58	24.37 ± 3.42	0.488	0.458	0.320	0.219	0.160	0.711
HR, bpm	71.97 ± 10.94	75.20 ± 14.77	72.07 ± 11.54	69.00 ± 14.63	0.340	0.973	0.378	0.364	0.108	0.371
SBP, mmHg	136.17 ± 14.34	131.07 ± 14.64	135.87 ± 20.92	132.97 ± 24.85	0.178	0.949	0.544	0.308	0.720	0.627
HTN, *n* (%)	20 (66.7)	23 (76.7)	20 (66.7)	17 (56.7)	0.399	1.000	0.434	0.399	0.104	0.434
DM, *n* (%)	7 (23.3)	10 (33.3)	11 (36.7)	15 (50.0)	0.390	0.260	0.032	0.787	0.190	0.297
COPD, *n* (%)	3 (10.0)	4 (13.3)	3 (10.0)	5 (16.7)	0.688	1.000	0.448	0.688	0.718	0.448
Known valvular disease, *n* (%)	1 (3.3)	1 (3.3)	0 (0)	2 (6.7)	1.000	0.313	0.554	0.313	0.554	0.150
ALT, U/L	20.50 ± 7.44	20.90 ± 10.98	23.17 ± 18.29	27.77 ± 18.70	0.869	0.464	0.055	0.563	0.089	0.339
Scr, μmol/L	76.10 ± 21.89	73.50 ± 21.07	73.77 ± 23.22	73.93 ± 14.42	0.641	0.690	0.653	0.963	0.926	0.973
eGFR, mL/(min*l.73 m^2^)	97.27 ± 16.18	101.63 ± 28.27	100.29 ± 30.73	94.37 ± 18.12	0.468	0.636	0.515	0.862	0.242	0.368
LDL, mmol/L	2.49 ± 0.69	2.32 ± 0.79	2.59 ± 0.83	2.29 ± 0.79	0.391	0.615	0.321	0.212	0.898	0.171
hs-CRP, mg/L	2.01 ± 1.74	3.38 ± 3.93	3.45 ± 4.12	6.31 ± 10.00	0.090	0.086	0.027	0.943	0.143	0.155
Coronary arteries	42	43	42	40	0.158	0.520	0.790	0.714	0.207	0.616
LAD, *n* (%)	16 (38.1)	22 (51.2)	20 (47.6)	18 (45.0)						
LCX, *n* (%)	8 (19.0)	11 (25.6)	9 (21.4)	6 (15.0)						
RCA, *n* (%)	18 (42.9)	10 (23.2)	13 (31.0)	16 (40.0)						

*F, female; M, male; BMI, body mass index; HR, heart rate; SBP, systolic blood pressure; HTN, hypertension; DM, diabetes mellitus; COPD, chronic obstructive pulmonary disease; ALT, alanine transaminase; Scr, serum creatinine; eGFR, estimated glomerular filtration rate; LDL, low-density lipoprotein; hs-CRP, high-sensitivity C-reactive protein; LAD, left anterior descending artery; LCX, left circumflex artery; RCA, right coronary artery*.

### Serum sST2 Level and Plaque Components in Volumetric Analysis of the Whole Lesion

As shown in Table 2, the relationships between the serum sST2 level and the absolute volumes or percentages of four different plaque components throughout the entire lesion were assessed. We found that patients with higher sST2 levels had a larger absolute NC volume (Quartile 4 vs. Quartile 1, 86.16 ± 59.71 vs. 45.10 ± 45.80 mm^3^, *P* = 0.001; Quartile 4 vs. Quartile 2, 86.16 ± 59.71 vs. 50.22 ± 42.56 mm^3^, *P* = 0.002) and a higher NC percentage (Quartile 4 vs. Quartile 1, 35.16 ± 9.82% vs. 23.21 ± 16.18%, *P* < 0.001; Quartile 4 vs. Quartile 2, 35.16 ± 9.82% vs. 22.50 ± 14.03%, *P* < 0.001; Quartile 4 vs. Quartile 3, 35.16 ± 9.82% vs. 25.04 ± 14.48%, *P* < 0.001). On the contrary, patients with higher sST2 levels had a lower DC percentage (Quartile 4 vs. Quartile 1, 7.23 ± 9.76% vs. 18.07 ± 22.13%, *P* = 0.005; Quartile 4 vs. Quartile 2, 7.23 ± 9.76% vs. 17.66 ± 19.89%, *P* = 0.003). No differences were observed in the other plaque characteristics, including the mean plaque burden, maximal plaque thickness, FT and FF components. The serum sST2 level was positively correlated with both the absolute NC volume (Pearson's *r* = 0.323, *P* < 0.001) and the NC percentage (Pearson's *r* = 0.425, *P* < 0.001). In addition, the serum sST2 level was mildly negatively correlated with the absolute DC volume (Pearson's *r* = −0.208, *P* = 0.007) and the DC percentage (Pearson's *r* = −0.275, *P* < 0.001) (Table 3).

**Table 2:** Serum sST2 level and plaque components in volumetric analysis of the whole lesion.

	**Quartile 1 (*N =* 30) <14.5 ng/mL**	**Quartile 2 (*N =* 30) 14.5–20.5 ng/mL**	**Quartile 3 (*N =* 30) 20.5–25.9 ng/mL**	**Quartile 4 (*N =* 30) >25.9 ng/mL**	***P*****-value**					
					**Quartile 2 vs. Quartile 1**	**Quartile 3 vs. Quartile 1**	**Quartile 4 vs. Quartile 1**	**Quartile 3 vs. Quartile 2**	**Quartile 4 vs. Quartile 2**	**Quartile 4 vs. Quartile 3**
Analyzed lesions, *n*	42	43	42	40						
Plaque volume, mm^3^	240.18 ± 209.36	217.95 ± 133.49	230.18 ± 181.63	220.45 ± 143.18	0.562	0.816	0.618	0.725	0.935	0.788
Mean plaque burden, %	60.34 ± 8.78	62.47 ± 7.71	60.86 ± 12.64	59.80 ± 9.35	0.238	0.829	0.787	0.480	0.160	0.666
Maximal plaque thickness, mm	2.57 ± 0.70	2.45 ± 0.74	2.41 ± 0.86	2.36 ± 0.50	0.421	0.352	0.110	0.844	0.512	0.718
Fibrous volume, mm^3^	73.79 ± 40.17	75.36 ± 43.05	74.62 ± 57.81	73.73 ± 46.89	0.862	0.939	0.995	0.947	0.870	0.939
Fibrous volume, %	35.32 ± 13.42	38.97 ± 13.24	37.50 ± 16.72	34.80 ± 9.91	0.211	0.512	0.841	0.656	0.107	0.374
Fibrous fatty volume, mm^3^	35.80 ± 19.47	35.49 ± 23.50	36.87 ± 30.42	37.34 ± 28.21	0.947	0.848	0.775	0.816	0.747	0.942
Fibrous fatty volume, %	18.25 ± 7.10	17.95 ± 7.15	18.18 ± 7.19	18.13 ± 7.26	0.848	0.964	0.937	0.885	0.914	0.973
Necrotic core volume, mm^3^	45.10 ± 45.80	50.22 ± 42.56	62.65 ± 69.86	86.16 ± 59.71	0.595	0.178	0.001	0.326	0.002	0.105
Necrotic core volume, %	23.21 ± 16.18	22.50 ± 14.03	25.04 ± 14.48	35.16 ± 9.82	0.829	0.588	<0.001	0.415	<0.001	<0.001
Dense calcium volume, mm^3^	74.21 ± 172.44	48.16 ± 78.62	39.11 ± 67.42	16.72 ± 26.96	0.376	0.225	0.039	0.570	0.017	0.051
Dense calcium volume, %	18.07 ± 22.13	17.66 ± 19.89	14.61 ± 18.40	7.23 ± 9.76	0.929	0.438	0.005	0.464	0.003	0.026

**Table 3:** Correlation between serum sST2 and plaque components in volumetric analysis of the whole lesion.

**Parameters**	**Pearson correlation**	***P*-value**
Plaque volume, mm^3^	−0.019	0.807
Mean plaque burden, %	0.018	0.818
Maximal plaque thickness, mm	−0.071	0.363
Fibrous volume, mm^3^	−0.059	0.446
Fibrous volume, %	−0.166	0.056
Fibrous fatty volume, mm^3^	0.088	0.257
Fibrous fatty volume, %	0.093	0.230
Necrotic core volume, mm^3^	0.323	<0.001
Necrotic core volume, %	0.425	<0.001
Dense calcium volume, mm^3^	−0.208	0.007
Dense calcium volume, %	−0.275	<0.001

### Serum sST2 Level and Plaque Components at the Level of the Minimal Lumen Area (MLA)

As shown in Table 4, there were no differences in the plaque components at the level of the minimal lumen area among the groups.

**Table 4:** Serum sST2 and plaque components at the level of the MLA.

	**Quartile 1 (*N =* 30) <14.5 ng/mL**	**Quartile 2 (*N =* 30) 14.5–20.5 ng/mL**	**Quartile 3 (*N =* 30) 20.5–25.9 ng/mL**	**Quartile 4 (*N =* 30) >25.9 ng/mL**	***P*****-value**					
					**Quartile 2 vs. Quartile 1**	**Quartile 3 vs. Quartile 1**	**Quartile 4 vs. Quartile 1**	**Quartile 3 vs. Quartile 2**	**Quartile 4 vs. Quartile 2**	**Quartile 4 vs. Quartile 3**
Analyzed lesions, *n*	42	43	42	40						
Eccentricity index	0.65 ± 015	0.68 ± 0.22	0.71 ± 0.21	0.72 ± 0.26	0.602	0.174	0.182	0.453	0.420	0.897
Plaque burden, %	79.11 ± 12.21	81.43 ± 10.49	77.31 ± 14.23	76.93 ± 15.13	0.378	0.561	0.497	0.150	0.132	0.911
Remodeling index	1.01 ± 0.16	0.98 ± 0.11	0.97 ± 0.15	0.97 ± 0.19	0.285	0.166	0.392	0.966	0.983	0.989
Maximal plaque thickness, mm	1.96 ± 0.47	1.91 ± 0.49	2.08 ± 0.56	1.86 ± 0.63	0.627	0.320	0.428	0.148	0.702	0.107
Fibrous area, mm^2^	3.24 ± 2.27	3.18 ± 1.89	2.94 ± 1.84	2.64 ± 1.88	0.899	0.538	0.222	0.574	0.209	0.483
Fibrous area, %	27.11 ± 16.02	26.26 ± 12.41	24.20 ± 14.14	27.45 ± 18.65	0.796	0.411	0.934	0.495	0.741	0.395
Fibrous fatty area, mm^2^	2.86 ± 2.35	2.29 ± 1.29	2.37 ± 1.97	2.14 ± 1.41	0.203	0.340	0.119	0.831	0.625	0.560
Fibrous fatty area, %	21.92 ± 12.91	21.06 ± 11.88	18.24 ± 11.26	19.20 ± 10.32	0.764	0.197	0.322	0.282	0.458	0.699
Necrotic core area, mm^2^	4.12 ± 2.28	4.00 ± 3.01	4.20 ± 3.21	3.33 ± 3.01	0.850	0.895	0.205	0.777	0.321	0.223
Necrotic core area, %	32.00 ± 15.17	33.24 ± 19.02	31.62 ± 20.14	28.99 ± 21.61	0.751	0.927	0.488	0.714	0.357	0.585
Dense calcium area, mm^2^	1.11 ± 2.02	1.77 ± 2.77	2.00 ± 3.08	1.93 ± 2.92	0.232	0.146	0.165	0.732	0.809	0.917
Dense calcium area, %	9.69 ± 15.78	14.24 ± 20.60	15.59 ± 23.80	17.15 ± 23.69	0.279	0.213	0.116	0.789	0.564	0.776

### Soluble ST2 and Plaque Calcification

**Figure 3:**
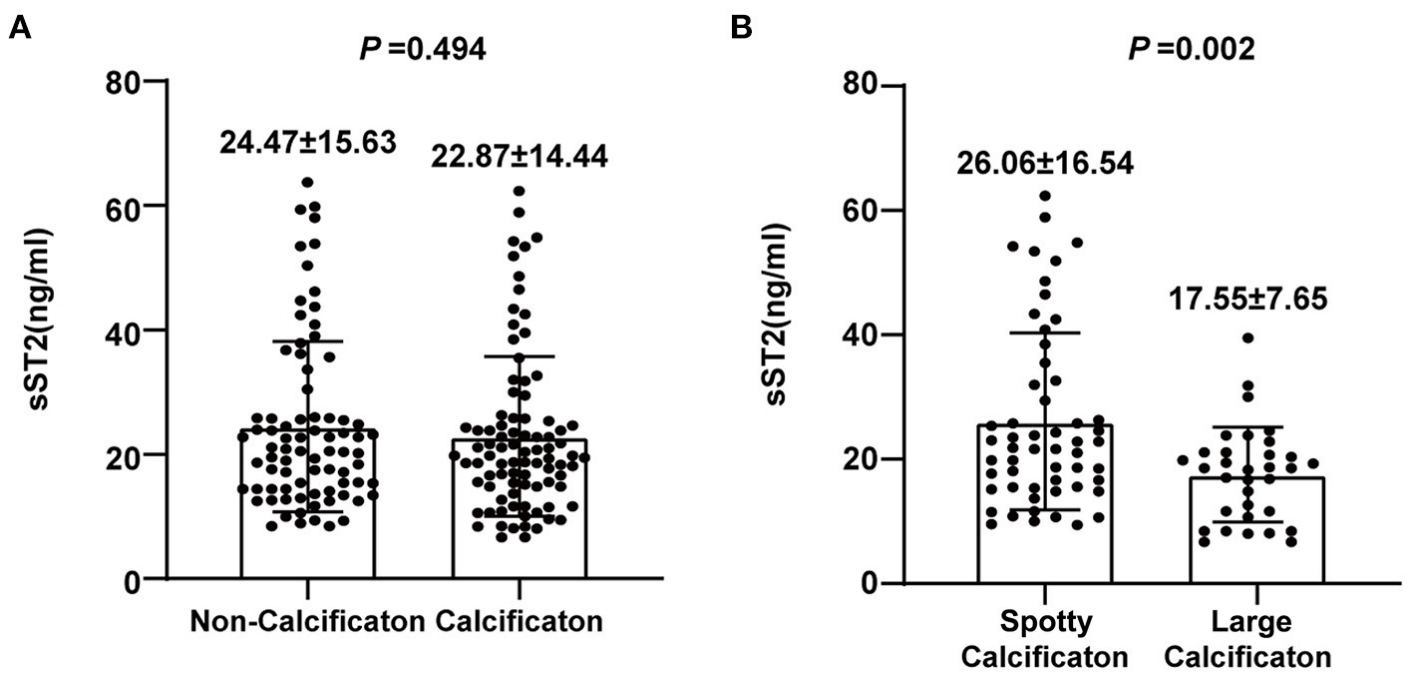
Analysis of the association between sST2 and plaque calcification. **(A)** No significant difference in serum sST2 levels was observed between the calcification and non-calcification groups. **(B)** Patients with spotty calcification had higher sST2 levels than those with large calcification.

Different types of plaque calcification play different roles in plaque vulnerability. To further clarify the correlation between the serum sST2 level and coronary plaque calcification, we divided patients into two groups based on the existence of plaque calcification. The results showed no significant difference in the serum sST2 levels (22.87 ± 14.44 vs. 24.47 ± 15.63 ng/mL, *P* = 0.494) between the calcification and non-calcification groups (Figure 3A). However, further division of the calcification group into two subgroups by the plaque calcification type revealed that patients with spotty calcification had higher sST2 levels than those with large calcification (26.06 ± 16.54 vs. 17.55 ± 7.65 ng/mL, *P* = 0.002) (Figure 3B).

## Discussion

The main findings of this study are as follows: (a) Among the four coronary plaque component phenotypes assessed by CCTA, the serum sST2 level was correlated with the NC and DC components. The volume of the NC component was larger in patients with higher serum sST2 levels, while that of the DC was decreased in these patients. (b) Further subgroup analysis revealed that patients with spotty calcification had higher serum sST2 levels than those with large calcification.

Previous studies have shown that inflammation plays an essential role in atherosclerosis, especially in NC formation, and NC has been proven to be tightly correlated with plaque vulnerability (23–29). As an important inflammatory factor, sST2 exerts proinflammatory effects and regulates the pathogenesis of atherosclerosis. A recent study by Zhang et al., as mentioned above, demonstrated that patients with ACS had higher serum sST2 levels than those with stable angina pectoris (SAP), which suggested that sST2 is potentially associated with plaque vulnerability (4). In our study, we further investigated the correlation between the serum sST2 level and plaque vulnerability by assessing the coronary plaque components in patients with non-ST-elevation ACS and revealed a positive correlation between the serum sST2 level and NC component in coronary lesions.

The role of calcification in plaque vulnerability remains controversial. Previous studies have reported a biphasic association between calcification and plaque vulnerability, as spotty calcification was more often found in ruptured plaques, while large calcification was more strongly related to stable plaques (30). Interestingly, patients with higher serum sST2 levels had smaller intraplaque calcification volumes in our study, and further subgroup analysis revealed that patients with spotty calcification had higher serum sST2 levels than those with large calcification. Furthermore, macrophages may play a role in the relationship between sST2 and calcification. Previous studies have demonstrated that sST2 may suppress the differentiation of macrophages toward the anti-inflammatory M2 phenotype (31). Proinflammatory M1 macrophages may facilitate microcalcification formation in plaque progression, leading to plaque rupture, while anti-inflammatory M2 macrophages are related to macrocalcification in plaque regression, suggesting plaque stability (32).

However, we did not observe associations between the serum sST2 level and plaque components at the level of the MLA. Perhaps the cross-sectional characteristics of the plaques at the level of the MLA were unable to reflect the characteristics of the whole plaque. Moreover, a similar conclusion was drawn in a previous study focused on carotid plaques, as no associations between serum sST2 levels and cross-sectional plaque characteristics were found on specimens from carotid endarterectomy (33).

In addition, we have observed that higher serum sST2 levels were correlated with older age in our study, which is consistent with previous studies (4, 34). Usually, aging is accompanied with alterations of cytokine expression toward a pro-inflammatory pattern (35–37). sST2, a modulator of the inflammatory response, might increase with age and exert a pro-inflammatory effect, while the underlying mechanisms need further study.

The current study does have some limitations. First, the study was observational in nature and was performed at a single center. Second, although this preliminary study has observed associations between serum sST2 levels and plaque vulnerability, only surrogate and not clinical endpoints were analyzed due to a relatively small sample size. Previous studies have demonstrated that elevation of serum sST2 levels were associated with poor prognosis in patients with myocardial infarction (MI) and heart failure (HF) (6–8). A larger sample size study is required in future to further investigate the predictive value of serum sST2 on long-term prognosis of patients with non-ST-elevation ACS. Third, although our study revealed that serum sST2 levels were correlated with NC and DC plaque components, the underlying mechanisms remain unclear. Previous study reported the role of sST2 in facilitating M1 macrophage polarization, which participated in the formation of spotty calcification and vulnerable plaque (31, 32, 38). However, the pathophysiological effects of sST2 on plaque vulnerability remain largely unknown, which need further mechanistic studies.

## Conclusions

Our study demonstrated that serum sST2 levels were correlated with different coronary plaque components in patients with non-ST-elevation ACS. Patients with higher serum sST2 levels had a larger NC plaque component. The serum sST2 level might be a useful predictive marker of plaque vulnerability in patients with non-ST-elevation ACS.

## Data Availability Statement

The original contributions presented in the study are included in the article/supplementary material, further inquiries can be directed to the corresponding author/s.

## Ethics Statement

The studies involving human participants were reviewed and approved by Institutional Review Board of Renji Hospital. The patients/participants provided their written informed consent to participate in this study.

## Author Contributions

SD and JP conceived and designed the study and amended the manuscript. GL, YQ, ZW, and FL acquired clinical data. QF and YY analyzed QAngioCT data. XS and JS executed the statistical analysis. GL and YQ drafted the manuscript. All authors contributed to the interpretation of the data and approved the final version of this manuscript.

## Conflict of Interest

The authors declare that the research was conducted in the absence of any commercial or financial relationships that could be construed as a potential conflict of interest.
